# Bis[5-(pyridin-2-yl-κ*N*)tetra­zolido-κ*N* ^1^]copper(II)

**DOI:** 10.1107/S1600536814002062

**Published:** 2014-02-05

**Authors:** Jian-Quan Li, Dan Mu, Meng-Bao Fan

**Affiliations:** a School of Mechatronic Engineering, China University of Mining and Technology (Xuzhou), Jiangsu 221116, People’s Republic of China; b Opto-Electronic Engineering College, Zaozhuang University, Shandong 277160, People’s Republic of China; c College of Chemistry Chemical Engineering and Materials Science, Zaozhuang University, Shandong 277160, People’s Republic of China

## Abstract

In the title complex, [Cu(C_6_H_4_N_5_)_2_], the Cu^II^ ion lies on an inversion center and is coordinated by two chelating 5-(pyridin-2-yl)tetra­zolide ligands in a slightly distorted square-planar coordination geometry. In the crystal, π–π stacking inter­actions, with centroid–centroid distances in the range 3.4301 (14)–3.4387 (13) Å, link the complex mol­ecules along [101].

## Related literature   

For background to coordination complexes, see: Lu *et al.* (2011 ▶); Yang *et al.* (2012 ▶).
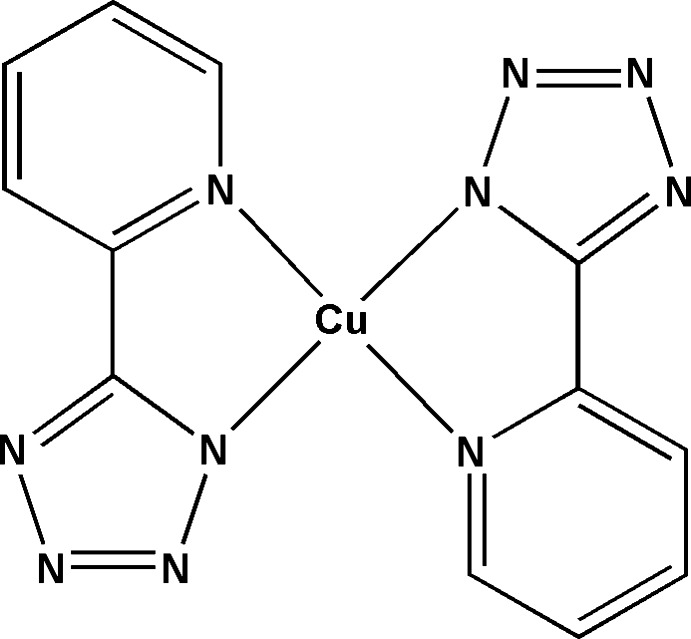


## Experimental   

### 

#### Crystal data  

[Cu(C_6_H_4_N_5_)_2_]
*M* *_r_* = 355.82
Monoclinic, 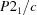

*a* = 5.5391 (9) Å
*b* = 13.128 (2) Å
*c* = 8.7950 (15) Å
β = 97.650 (3)°
*V* = 633.88 (18) Å^3^
*Z* = 2
Mo *K*α radiation
μ = 1.74 mm^−1^
*T* = 291 K
0.44 × 0.35 × 0.30 mm

#### Data collection  

Rigaku R-AXIS RAPID diffractometer
Absorption correction: multi-scan (*ABSCOR*; Higashi, 1995 ▶) *T*_min_ = 0.486, *T*_max_ = 0.593
3328 measured reflections
1241 independent reflections
1063 reflections with *I* > 2σ(*I*)
*R*_int_ = 0.037

#### Refinement  

*R*[*F* ^2^ > 2σ(*F* ^2^)] = 0.034
*wR*(*F* ^2^) = 0.087
*S* = 1.03
1241 reflections
106 parameters
H-atom parameters constrained
Δρ_max_ = 0.48 e Å^−3^
Δρ_min_ = −0.42 e Å^−3^

### 

Data collection: *RAPID-AUTO* (Rigaku, 1998 ▶); cell refinement: *RAPID-AUTO*; data reduction: *CrystalStructure* (Rigaku/MSC, 2006 ▶); program(s) used to solve structure: *SHELXS97* (Sheldrick, 2008 ▶); program(s) used to refine structure: *SHELXL97* (Sheldrick, 2008 ▶); molecular graphics: *ORTEPII* (Johnson, 1976 ▶); software used to prepare material for publication: *SHELXL97*.

## Supplementary Material

Crystal structure: contains datablock(s) I. DOI: 10.1107/S1600536814002062/lh5686sup1.cif

Structure factors: contains datablock(s) I. DOI: 10.1107/S1600536814002062/lh5686Isup2.hkl

CCDC reference:

Additional supporting information:  crystallographic information; 3D view; checkCIF report

## Figures and Tables

**Fig. 1.**
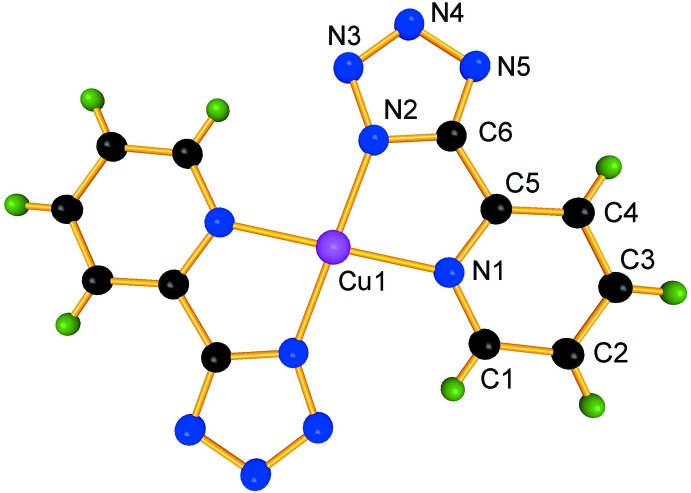
The molecular structure with ellipsoids drawn at the 30% probability level. Unlabeled atoms are related by the symmetry operator (-x, -y+1, -z+1).

**Fig. 2.**
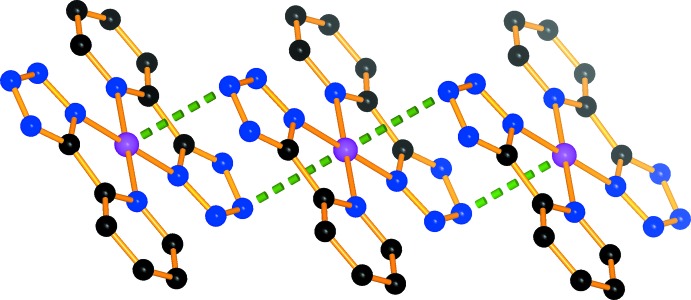
Part of the crystal structure indicating π–π stacking interactions along [101] and dashed lines to show closest intermolecular Cu···N contacts.

## supplementary crystallographic information

### 1. Comment

Coordination complexes (polymers) have drawn broad attention in recent decades due to their promising applications in catalysis, sensing and gas adsorption/separation (Yang *et al.*, 2012; Lu *et al.*, 2011). Despite several investigations, a detailed analysis of single crystal structures of coordination complexes is also of importance for the study of specific bonding between supramolecules in the solid state.

We report here a coordination complex, formulated as Cu(pytz)_2_ (pytz = 5-(pyridin-2-yl)tetrazolide). The molecular structure of the title compound is shown in Fig. 1. The Cu^II^ ion is located on an inversion center. The pytz ligand coordinates to the Cu^II^ ion *via* two symmetry related pyridine N atoms and two symmetry related tetrazolide N atoms. The Cu—N bond distances are 1.956 (2) and 2.021 (2) Å. In the crystal, π–π stacking interactions with centroid–centroid distances of 3.4386 (13)Å for Cg1···Cg3(-x+1, -y+1, -z+1), 3.4387 (14)Å for Cg2···Cg3 (-1+x, y, z) and 3.4301 (14)Å for Cg3–Cg3 (-1+x, -1+y, -1+z) link the complex molecules along [101]. Cg1, Cg2 and Cg3 are the centroids of the Cu1/N1/C5/C6/N2, Cu1/N1^i^/C5^i^/C6^i^/N2^i^ (symmetry code (i):-x, -y+1, -z+1) and N2/N3/N4/N5/N6 rings, respectively. These stacking interactions allow for intermolecular Cu···N contacts of 2.993 (1)Å (Fig. 2).

### 2. Experimental

The title complex was synthesized by the addition of CuNO_3_ (2 mmol) to an ethanol solution of Hpytz (4 mmol). The mixed solution was allowed to evaporated solwly at room temperature, and blue prismatic crystals were isolated in about 15 days. Analysis calculated for C_12_H_8_N_10_Cu: C 40.51, H 2.27, N 39.37%; Found: C 40.48, H 2,21, N 39.30%.

### 3. Refinement

The H atoms on carbon were placed in calculated positions [C—H = 0.93 Å (aromatic), and *U*_iso_(H) = 1.2*U*_eq_(C_aromatic_)] using a riding model approximation.

### Crystal data

**Table:**

[Cu(C_6_H_4_N_5_)_2_]	*F*(000) = 358
*M**_r_* = 355.82	*D*_x_ = 1.864 Mg m^−^^3^
Monoclinic, *P*2_1_/*c*	Mo *K*α radiation, λ = 0.71073 Å
*a* = 5.5391 (9) Å	Cell parameters from 1641 reflections
*b* = 13.128 (2) Å	θ = 2.3–25.1°
*c* = 8.7950 (15) Å	µ = 1.74 mm^−^^1^
β = 97.650 (3)°	*T* = 291 K
*V* = 633.88 (18) Å^3^	Block, blue
*Z* = 2	0.44 × 0.35 × 0.30 mm

### Data collection

**Table:**

Rigaku R-AXIS RAPID diffractometer	1241 independent reflections
Radiation source: fine-focus sealed tube	1063 reflections with *I* > 2σ(*I*)
Detector resolution: 10 pixels mm^-1^	*R*_int_ = 0.037
ω scan	θ_max_ = 26.0°, θ_min_ = 2.8°
Absorption correction: multi-scan (*ABSCOR*; Higashi, 1995)	*h* = −6→6
*T*_min_ = 0.486, *T*_max_ = 0.593	*k* = −9→16
3328 measured reflections	*l* = −10→10

### Refinement

**Table:**

Refinement on *F*^2^	0 restraints
Least-squares matrix: full	Hydrogen site location: inferred from neighbouring sites
*R*[*F*^2^ > 2σ(*F*^2^)] = 0.034	H-atom parameters constrained
*wR*(*F*^2^) = 0.087	*w* = 1/[σ^2^(*F*_o_^2^) + (0.055*P*)^2^] where *P* = (*F*_o_^2^ + 2*F*_c_^2^)/3
*S* = 1.03	(Δ/σ)_max_ < 0.001
1241 reflections	Δρ_max_ = 0.48 e Å^−^^3^
106 parameters	Δρ_min_ = −0.42 e Å^−^^3^

### Special details

**Table:**

Geometry. All e.s.d.'s (except the e.s.d. in the dihedral angle between two l.s. planes) are estimated using the full covariance matrix. The cell e.s.d.'s are taken into account individually in the estimation of e.s.d.'s in distances, angles and torsion angles; correlations between e.s.d.'s in cell parameters are only used when they are defined by crystal symmetry. An approximate (isotropic) treatment of cell e.s.d.'s is used for estimating e.s.d.'s involving l.s. planes.

### Fractional atomic coordinates and isotropic or equivalent isotropic displacement parameters (Å^2^)

**Table:**

	*x*	*y*	*z*	*U*_iso_*/*U*_eq_	
Cu1	0.0000	0.5000	0.5000	0.03232 (18)	
N3	0.4142 (4)	0.54997 (16)	0.3062 (2)	0.0405 (5)	
N5	0.5268 (4)	0.39183 (15)	0.2804 (2)	0.0396 (5)	
N1	0.0300 (3)	0.34667 (17)	0.49523 (18)	0.0316 (5)	
C5	0.2056 (4)	0.31234 (17)	0.4168 (2)	0.0303 (5)	
N2	0.2605 (4)	0.49021 (12)	0.3709 (2)	0.0325 (5)	
C6	0.3376 (4)	0.39545 (17)	0.3530 (2)	0.0311 (5)	
C4	0.2523 (4)	0.21184 (17)	0.3970 (3)	0.0389 (6)	
H4	0.3775	0.1913	0.3433	0.047*	
N4	0.5743 (5)	0.49081 (14)	0.2522 (3)	0.0430 (6)	
C1	−0.1079 (4)	0.27709 (18)	0.5552 (3)	0.0376 (6)	
H1	−0.2307	0.2990	0.6099	0.045*	
C3	0.1085 (5)	0.14121 (19)	0.4590 (3)	0.0426 (6)	
H3	0.1346	0.0719	0.4471	0.051*	
C2	−0.0724 (5)	0.17453 (18)	0.5379 (3)	0.0421 (6)	
H2	−0.1714	0.1279	0.5797	0.051*	

### Atomic displacement parameters (Å^2^)

**Table:**

	*U*^11^	*U*^22^	*U*^33^	*U*^12^	*U*^13^	*U*^23^
Cu1	0.0308 (3)	0.0254 (3)	0.0450 (3)	0.00163 (14)	0.02087 (19)	0.00056 (15)
N3	0.0396 (12)	0.0350 (12)	0.0520 (13)	−0.0013 (10)	0.0251 (10)	0.0024 (10)
N5	0.0390 (11)	0.0353 (11)	0.0491 (12)	−0.0004 (9)	0.0231 (9)	−0.0020 (9)
N1	0.0297 (10)	0.0279 (11)	0.0393 (11)	0.0002 (7)	0.0128 (9)	0.0002 (7)
C5	0.0277 (11)	0.0298 (12)	0.0351 (11)	−0.0005 (9)	0.0105 (9)	−0.0012 (9)
N2	0.0316 (11)	0.0285 (11)	0.0410 (12)	−0.0005 (7)	0.0180 (9)	0.0007 (7)
C6	0.0301 (12)	0.0283 (11)	0.0369 (12)	0.0000 (9)	0.0119 (9)	−0.0027 (9)
C4	0.0399 (13)	0.0331 (13)	0.0473 (14)	0.0015 (10)	0.0190 (11)	−0.0042 (10)
N4	0.0422 (13)	0.0370 (13)	0.0562 (14)	−0.0011 (8)	0.0298 (11)	−0.0010 (9)
C1	0.0347 (12)	0.0342 (13)	0.0470 (14)	−0.0003 (10)	0.0176 (11)	0.0009 (10)
C3	0.0496 (15)	0.0266 (12)	0.0556 (15)	0.0015 (11)	0.0216 (13)	−0.0033 (11)
C2	0.0461 (14)	0.0324 (14)	0.0512 (15)	−0.0061 (11)	0.0193 (12)	0.0030 (11)

### Geometric parameters (Å, º)

**Table:**

Cu1—N2	1.956 (2)	C5—C4	1.360 (3)
Cu1—N2^i^	1.956 (2)	C5—C6	1.466 (3)
Cu1—N1^i^	2.021 (2)	N2—C6	1.331 (3)
Cu1—N1	2.021 (2)	C4—C3	1.381 (3)
N3—N4	1.314 (3)	C4—H4	0.9300
N3—N2	1.339 (3)	C1—C2	1.372 (3)
N5—C6	1.299 (3)	C1—H1	0.9300
N5—N4	1.355 (2)	C3—C2	1.364 (4)
N1—C1	1.343 (3)	C3—H3	0.9300
N1—C5	1.343 (3)	C2—H2	0.9300
			
N2—Cu1—N2^i^	180.0	N5—C6—N2	112.6 (2)
N2—Cu1—N1^i^	98.42 (7)	N5—C6—C5	129.6 (2)
N2^i^—Cu1—N1^i^	81.58 (7)	N2—C6—C5	117.8 (2)
N2—Cu1—N1	81.58 (7)	C5—C4—C3	118.1 (2)
N2^i^—Cu1—N1	98.42 (7)	C5—C4—H4	120.9
N1^i^—Cu1—N1	180.0	C3—C4—H4	120.9
N4—N3—N2	107.76 (19)	N3—N4—N5	110.1 (2)
C6—N5—N4	104.1 (2)	N1—C1—C2	121.8 (2)
C1—N1—C5	117.5 (2)	N1—C1—H1	119.1
C1—N1—Cu1	128.11 (16)	C2—C1—H1	119.1
C5—N1—Cu1	114.33 (15)	C2—C3—C4	119.1 (2)
N1—C5—C4	123.7 (2)	C2—C3—H3	120.4
N1—C5—C6	112.3 (2)	C4—C3—H3	120.4
C4—C5—C6	124.1 (2)	C3—C2—C1	119.8 (2)
C6—N2—N3	105.40 (19)	C3—C2—H2	120.1
C6—N2—Cu1	113.75 (15)	C1—C2—H2	120.1
N3—N2—Cu1	140.20 (15)		

Symmetry code: (i) −*x*, −*y*+1, −*z*+1.
